# A novel exopolysaccharide produced by *Lactobacillus coryniformis* NA-3 exhibits antioxidant and biofilm-inhibiting properties *in vitro*

**DOI:** 10.29219/fnr.v64.3744

**Published:** 2020-04-03

**Authors:** Xiaoqing Xu, Qing Peng, Yuwei Zhang, Dandan Tian, Pengbo Zhang, Ying Huang, Lan Ma, Yu Qiao, Bo Shi

**Affiliations:** Feed Research Institute, Chinese Academy of Agricultural Sciences, Beijing, PR China

**Keywords:** *Lactobacillus coryniformis* NA-3, exopolysaccharide, antioxidant, anti-biofilm, inhibition, dispersion

## Abstract

**Background:**

Exopolysaccharides (EPSs) secreted from lactic acid bacteria are carbohydrate polymers with reported biological activities. In this study, we extracted and characterized the composition as well as antioxidant and biofilm-inhibitory properties of EPS from *Lactobacillus coryniformis* NA-3 isolated from northeast Chinese sauerkraut (Suan Cai).

**Methods:**

*Lactobacillus coryniformis* NA-3 was identified with 16S rDNA amplification and Neighbor Joining (NJ) phylogenetic analysis. EPS derived from *Lactobacillus coryniformis* NA-3 (EPS-NA3) was analyzed, including compositions by high-performance liquid chromatography (HPLC), functional groups by Fourier-transform infrared spectroscopy (FT-IR) and glycosidic bond configuration by Hydrogen-1 Nuclear Magnetic Resonance (^1^H NMR). Antioxidant activity of EPS was evaluated with hydroxyl and superoxide radical-scavenging. Anti-biofilm activities of EPS-NA3 were checked through inhibition and dispersion.

**Results:**

The monosaccharide composition of EPS included α-rhamnose, α-mannose, α-galactose, and α-glucose in a ratio of 2.6:1.0:5.0:3.3. The free radical-scavenging abilities of EPS-NA3 were 37.77% ± 1.56% and 78.87% ± 3.07% on hydroxyl and superoxide reactive oxygen species respectively. Moreover, EPS-NA3 attenuated the formation of *Bacillus cereus* and *Salmonella typhimurium* biofilms by inhibition ratios of approximately 80% and 40% respectively. Additionally, treatment with EPS-NA3 dispersed established biofilms of *B. cereus* and *S. typhimurium* by approximately 90% and 20% respectively.

**Conclusion:**

These results suggest that EPS-NA3 may be developed as antioxidant and anti-biofilm agents for industrial and clinical applications due to its capacity of scavenging free radicals, inhibition of bacterial biofilm formation, and dispersion of established biofilms.

## Popular scientific summary

- The first report characterizing a novel exopolysaccharide (EPS) from *Lactobacillus coryniformis* NA-3, isolated from Northeast Chinese sauerkraut (Suan Cai).
- EPS derived from *Lactobacillus coryniformis* NA-3 is a heteropolysaccharide exhibited antioxidant activity.
- EPS derived from *Lactobacillus coryniformis* NA-3 inhibits pathogen biofilm formation or disperses biofilm.

Lactic acid bacteria (LAB) can produce exopolysaccharides (EPSs) on bacterial cell wall to form a capsule, or secreted in the environment as a viscous slime (1–3) for retaining moisture to protect bacteria or for scavenging external nutrients. EPSs are long-chain carbohydrate polymers linked by repeating monosaccharide units and classified into homopolysaccharides (HoPSs) and heteropolysaccharides (HePSs) according to monosaccharide composition (4). Homopolysaccharides mainly include glucans and fructans, occasionally galactans (5). HePSs are produced by a large variety of mesophilic (*Lactobacillus casei, Lb. rhamnosus*, etc.) and thermophilic (*Lb. acidophilus, Lb. helveticus*, etc.) LAB strains (6). Interest in EPSs produced by *Lactobacillus* spp. has increased due to potential economic reasons of replacing natural gums (7). Bacterial EPSs have shown improvements, compared with eukaryotic polysaccharides, in rheological characteristics and stability while also reducing the requirement for arable land necessary for their production (8).

Over the past decades, several studies have examined the physical properties of LAB-derived EPSs, such as their individual characteristics as biological agents for thickening, gelatinization, stabilization, and emulsification in food industry applications (9). In addition, EPSs from LAB are reported to provide health benefits, such as anti-tumor, immune-stimulating, and antioxidant activities as well as inhibition of bacterial growth and biofilm production (10–12). In particular, antioxidant and anti-biofilm activities have attracted increasing research attention as desirable properties to explore for medical, pharmaceutical, and industrial applications. An increase in oxidative stress is one factor that underlies the pathology of many diseases, such as diabetes, cancer, liver diseases, and several chronic degenerative diseases (13).

LAB-derived EPSs have been shown to enhance cellular defense mechanisms through antioxidant activity that reduces the oxidative damage caused by reactive oxygen species and free radicals (14, 15). Similarly, EPSs from LAB have been tested for biofilm inhibition to aid in the prevention of some clinical diseases caused by pathogenic bacteria, such as infections caused by *Staphylococcus epidermidis, Pseudomonas aeruginosa, Vibrio cholerae*, and *Yersinia* (16). Many EPS-producing *Lactobacillus* species, such as *Lactobacillus helveticus, L. plantarum* (17), *L. fermentum* (18), and *L. acidophilu*s (19) have also shown anti-biofilm activity. Interference with biofilm formation by pathogenic bacteria may comprise a large part of the antimicrobial effects of EPSs (12) which is important for preventing chronic and recurrent infections.

EPS-producing LAB have been isolated from diverse sources, most often from fermented foods (3, 20). Northeastern Chinese sauerkraut (or Suan Cai) is a traditional fermented food with a complex microbial community. *Lactobacillus* spp., including *L. plantarum, L. casei* (21), and *L. coryniformis* (22) are relatively abundant. Among Lactobacilli, *L. coryniformis* has been studied less than other species, although it is typically found in fermented vegetable products (23). A safety assessment of *L. coryniformis* CECT 5711 revealed no deleterious enzymatic activities for this species, although it exhibits an intrinsic antibiotic resistance profile (24). *L. coryniformis* CECT 5711 has also been reported to display antimicrobial activity (24) and is used as a probiotic strain to enhance intestinal function in healthy adults (25). However, the physical properties and biological activities of EPSs isolated from *L. coryniformis* have not been studied yet; in light of its uses as a probiotic, this species may prove to be a valuable source as an EPS-producing strain, safe for consumption and medical applications.

In the present work, we describe the isolation of EPS-producing strain *L. coryniformis* NA-3, extraction (by ethanol precipitation) and characterization of the EPS composition (with Fourier-transform infrared spectroscopy [FT-IR] and high-performance liquid chromatography [HPLC] analysis), and subsequent evaluation of its antioxidant and anti-biofilm activities. Our findings indicate that EPS produced by this LAB strain could serve as a substantial source of food production and pharmaceuticals, and has the potential of developing as a nutraceutical.

## Materials and methods

### Screening and identification of EPS-producing LAB

For isolation of LAB, (Suan Cai) brine was serially diluted in phosphate buffer solution (PBS; pH = 7.4) and plated on De Man, Rogosa, and Sharpe (MRS) (Solarbio, Beijing, China) agar medium with 0.3% CaCO_3_ (Xilong, Guangdong, China). The plates were incubated for 24–48 h at 37°C under anaerobic conditions. EPS-producing LAB strains were first screened by transparent zones or haloes showing calcium solubilization, and then viscid, ‘ropy’ colonies were isolated from calcium solubilizing colonies (26, 27). A single colony from each strain was inoculated into 10 mL of fresh MRS medium, and incubated at 37°C without shaking. Bacterial suspensions were centrifuged and the cell pellets were used for total DNA extraction with TIANamp Bacteria DNA Kit (Tiangen Biotech, Beijing, China). The 16S rDNA amplification was carried out with primers 27F (5’-AGTTTGATCMTGGCTCAG-3’) and 1492R (5’-TACGGYTACCTTGTTACGACTT-3’) using the following polymerized chain reaction (PCR) program: denaturation at 94°C for 3 min, 35 cycles at 94°C for 30 s, annealing at 55°C for 30 s, and extension at 72°C for 2 min, followed by a final extension cycle at 72°C/10 min. The PCR products were sent to Sango Biotech (Shanghai, China) for sequencing, and the sequences were identified using the NCBI non-redundant nucleotide BLAST (http://blast.ncbi.nlm.nih.gov). Subsequently, multiple sequence alignment (GenBank: EU626008.1, EU626013.1, MF629001.1, CP044506.1, NR_113175.1, FJ429977.1, NR_109004.1, EU626020.1, NR_117812.1, EU626019.1, EU331258.1, NR_118877.1, KX503225.1, FJ749472.1, and NR_040783.1) and Neighbor Joining (NJ) phylogenetic analysis were conducted using Mega 5.1. All isolates were stored at −80°C in medium with 30% glycerol (final concentration).

### Growth conditions of bacterial strains

The pathogenic bacterial strains used for biofilm inhibition and dispersion assays were *Bacillus cereus* (CICC 21261) (Gram-positive) and *Salmonella typhimurium* (CICC 22956/ATCC 14028) (Gram-negative), purchased from the China Center of Industrial Culture Collection.

*L. coryniformis* NA-3 was activated anaerobically in MRS broth at 37°C. *S. typhimurium* was grown in Tryptic Soy Broth (TSB) medium containing 1.7% tryptone (OXOID, Basingstoke, England), 0.3% peptone (Aoboxing, Beijing, China), 0.5% NaCl (Xilong, Guangdong, China), 0.25% K_2_HPO_4_ (Xilong, Guangdong, China), and 0.25% glucose (Xilong, Guangdong, China). *B. cereus* was cultivated in Nutrient Broth (Aoboxing, Beijing, China).

### EPS extraction from L. coryniformis NA-3

As described by Waśko et al. (28) and Yang et al. (29), the hot water extraction method was used to extract EPS from *L. coryniformis* NA-3 with minor modifications. *L. coryniformis* NA-3 was grown in MRS agar medium at 37°C for 24 h under anaerobic conditions. Then colony of *L. coryniformis* NA-3 was transferred to fresh MRS agar medium and incubated without shaking for 24 h. *L. coryniformis* NA-3 suspension was then inoculated at a 1/100 (v/v) dilution to 100 mL and 2,250 mL (750 mL × 3) of MRS medium successively and cultured at 37°C for 48 h under anaerobic conditions.

Following incubation, 2,250 mL of *L. coryniformis* NA-3 culture suspension was centrifuged at 12,000*×g* for 10 min and cells were washed twice with 0.9% NaCl. Then cells were treated with 80°C distilled water for about 20 h. After centrifugation (12,000*×g* for 20 min at 4°C), sediments were removed and the supernatant was retained as EPS solution. The EPS was precipitated by adding a three-fold volume of chilled ethanol, and the suspension was left to stand at 4°C for 4 days. Crude EPS was obtained after centrifugation at 12,000*×g* for 20 min, then resuspended in distilled water with 14% trichloroacetic acid (TCA) (Fuchen, Tianjin, China) and kept at 4°C for 24 h. Soluble proteins were removed after centrifugation at 12,000*×g* for 20 min at 4°C; supernatant was dialyzed for 3 days against distilled water (MWCO 7,000 Da, DL BioChem, USA), then concentrated and lyophilized, thus resulting in isolated polysaccharide.

### Molecular weight (Mw) determination of EPS

The uniformity of EPS was determined with a Waters 2695 HPLC system (Milford, MA, USA) equipped with a TSK gel GMPW XL column (300 mm × 7.8 mm, Tosoh Corp., Tokyo, Japan) and a refractive index detector. EPS solution (5 mg/mL), 10 μL, was injected and eluted with re-distilled water at a flow rate of 1 mL/min. The linear regression of dextran standards (Sigma-Aldrich, St. Louis, MO, USA) was calibrated to calculate the molecular weight of EPS.

### Determination of total sugar content

The phenol–sulfuric acid method was used to determine total carbohydrate using a 96-well plate and glucose as the standard (30). In brief, 20-μL EPS (1 mg/mL) or glucose solution and 20-μL phenol (5% water solution) were mixed in a well, 100-μL sulfuric acid was added to the mixture, and the mixture was measured at a wavelength of 480 nm using a microplate reader (BioTek Instruments, Inc. Winosky, VT, USA) after incubation for 30 min at 25°C.

### Determination of EPS monosaccharide composition

Monosaccharide composition of EPS was determined by hydrolyzing the sample, which was then neutralized with BaCO_3_ and concentrated by rotary evaporation after hydrolysis in 1-M sulfuric acid in 100°C water bath for 3 h. The monosaccharide solution was eluted by acetonitrile and water (9:1, v:v ) with a Shodex HILICpak VG-50 4E (250 mm × 4.6 mm, Shodex, Japan) using HPLC (Waters 2695, Milford, MA, USA) equipped with an evaporative light scattering detector (6100 Chromachem, ESA lnc.). Rhamnose, arabinose, mannose, galactose, and glucose were used as sugar standards (Tokyo Chemical Industry, Japan).

### Infrared (FT-IR) spectroscopic analysis of EPS

The potassium bromide (KBr) pellet pressing method was used in FT-IR to analyze the chemical composition of EPS over a wavelength range of 4,000 cm^−1^ to 400 cm^−1^.

### Nuclear magnetic resonance (NMR) spectroscopy analysis of the EPS

NMR spectra of EPS were measured on a Bruker Avance-500 spectrometer (Bruker Corporation, Karlsruhe, Germany). The sample was dissolved in D_2_O (>99.0%) and the internal calibration standard was MeOH-d4 (δ_H_ 3.31 and 4.87).

### Antioxidant activity of EPS

#### Hydroxyl radical-scavenging activity

The hydroxyl radical-scavenging activity of EPS was determined according to the method described by Wu et al. (31), with few modifications. In brief, PBS (20 mM, pH 7.4), 50 μL; 12.5-mM 1,10-phenanthroline solution, 25 μL; 2.5-mM FeSO_4_ solution, 25 μL; and 20-mM H_2_O_2_, 25 μL were added successively to each well of 96-well plates and mixed thoroughly. Then 100-μL EPS aliquots at various concentrations were added to the mixture, incubated at 37°C for 1 h, and immediately measured at 536 nm. Ascorbic acid was used as a positive control. Each experiment was performed in triplicate.

Hydroxyl radical-scavenging activity was expressed as the following formula:

Scavenging activity (%) = (As – Ac)/(Ao – Ac) × 100

where ‘As’ is the absorbance of the sample in the presence of different concentrations of EPS, ‘Ac’ is the absorbance of the sample in the absence of EPS, and ‘Ao’ is the absorbance of the sample without both EPS and H_2_O_2_.

#### Superoxide radical-scavenging activity

The superoxide radical-scavenging activity of EPS was determined following the protocols of Zhang (32) with minor modifications. An aliquot of 50 μL of Tris-HCl buffer (pH 8.0, 150 mM) was mixed with 25 μL of pyrogallol (1.50 mM, dissolved in 10-mM HCl) and 100-μL EPS aliquots at various concentrations; ascorbic acid was used as a positive control. The mixture was incubated at 25°C for 30 min after thorough mixing, and the absorbance of the mixture was measured at 325 nm. Each experiment was performed in triplicate.

Scavenging of superoxide radicals generated by pyrogallol autoxidation was calculated as follows:

Scavenging activity (%) = [1 – (A11 – A10)/(A01 – A00)]

where ‘A00’ is the absorbance of the sample in the absence of EPS and pyrogallol, ‘A01’ is the absorbance of the sample containing pyrogallol but no EPS, ‘A10’ is the absorbance of the sample containing EPS but no pyrogallol, and ‘A11’ is the absorbance of the sample containing EPS and pyrogallol.

### Anti-biofilm activities of EPS

#### Inhibition activity

The biofilm inhibition assay was performed as follows: *B. cereus* and *S. typhimurium* suspensions were inoculated in a 24-well plate (Costar, Corning, USA) with sterile EPS, at a final concentration of 500 μg/mL. Wells without EPS were simultaneously used as blank controls. A rectangular microscope cover glass (1 cm in diameter) was inserted into every well and the 24-well plate was incubated at 37°C for 24 h. The glass slides were carefully washed with PBS (pH 7.4) and biofilms that adhered to the slides were observed using a fluorescence microscope (Carl Zeiss Axio Vert.A1, Jena, Germany) after staining with 0.01% acridine orange solution for 5 min.

After qualitative analysis with fluorescence microscopy, quantitative measurement was done using a 96-well plate (Costar, Corning, USA) according to previously published methods (17, 33). *B. cereus* and *S. typhimurium* were added individually to the 96-well plate with different concentrations of EPS. The final concentrations of the EPS were 500, 250, 125, 62.50, and 31.25 μg/mL in sterile PBS (pH = 7.4). Negative controls lacked EPS and blank controls were only with PBS without any bacterial strains. The plates with *B. cereus* and *S. typhimurium* were incubated at 37°C for 24 h. The suspension in each well was removed and the microtiter wells were washed for four times with PBS to remove non-adherent cells. Biofilms were stained with MTT solution (prepared with the corresponding medium) and incubated in dark for 3 h at 37°C (34, 35). Dimethyl sulfoxide (DMSO) was used to rinse after removal of MTT solution. Optical density of each well was measured at 490 nm (OD_490_) using a Microplate Reader (BioTek, USA). The inhibition by EPS was inversely proportional to the OD_490_ value and the results were expressed using inhibition ratios.

#### Dispersion activity

The general procedures of biofilm dispersion experiments were conducted as follows: Biofilms of *B. cereus* and *S. typhimurium* were first inoculated in a 24-well plate (Costar, Corning, USA), as described above for inhibition assay, with a rectangular microscope cover glass added to each well, and incubated at 37°C for 24 h. The glass slides with biofilms were carefully washed with PBS (pH 7.4) and placed into another 24-well plate filled with fresh culture medium with or without EPS (dissolved with PBS, final concentration of 500 μg/mL), and incubated at 37°C for 24 h. The glass slides were observed under a fluorescence microscope (Carl Zeiss Axio Vert.A1, Jena, Germany) after careful washing and then staining with 0.01% acridine orange for 5 min.

However, for this assay, the efficacy of EPS in biofilm dispersion was determined after biofilm formation. As mentioned in the section ‘*Inhibition activity*’, after formation of biofilms, the bacterial suspensions were removed and the microtiter wells were washed for four times with PBS to remove non-adherent cells. Then fresh medium with different concentrations of EPS was added to each well (final concentrations were 500, 250, 125, 62.50, and 31.25 μg/mL). Negative and blank controls were as mentioned above. After incubation at 37°C for 24 h, the subsequent procedures were the same as described in the section ‘*Inhibition activity*’.

### Statistical analysis

All results were shown as mean ± standard deviation (SD) and data were analyzed by one-way ANOVA and Duncan’s test using SPSS software, version 17.0; *P* < 0.05 was used to identify statistically significant differences. All experiments included three biological replicates.

## Results

### Screening and identification of EPS-producing L. coryniformis

**Fig. 1:**
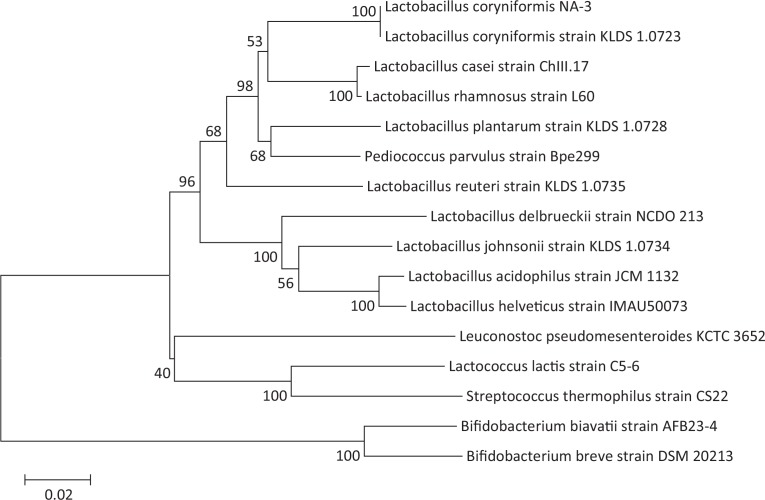
Neighbor joining-dendrogram showing the phylogenetic relationship between16S rDNA nucleotide sequences of *Lactobacillus* spp., obtained from the GenBank database, and *Lactobacillus coryniformis* NA-3 16S sequence, isolated in this study.

Initially, 247 LAB strains were screened from Suan Cai fermentation brine based on colony morphology and the presence of haloes in the medium indicating calcium solubilization. A total of 39 Gram-positive and catalase negative strains with viscid, mucoid colonies were streaked to isolation and identified by Sanger sequencing to determine genus and species. Among these 39 strains, BLAST searches of 16S rRNA sequence showed that the collections comprised the following: 2 *Pediococcus ethanolidurans*, 1 *Pediococcus parvulus*, 14 *L. coryniformis*, 16 *L. plantarum*, 3 *L. brevis*, 1 *L. sake sub sp. sakei*, 1 *L. paracasei*, and 1 *L. harbinensis*. One particularly viscid or ropy strain, designated *L. coryniformis* NA-3, was used for further experiments. To confirm the phylogenetic relationship of this strain with other LAB, we constructed a phylogenetic tree using an alignment and distance matrix of 16S sequences from NA-3 with those of LAB and bifido bacteria available in public databases (Fig. 1). Branching patterns showed that NA-3 was closely related to *L. coryniformis* strain KLDS 1.0723 (>99%), with which it clustered apart from other species of this genus, thus confirming the membership of NA-3 in the *L. coryniformis* species.

### EPS-NA3 is a heteropolysaccharide composed of four monomers

**Fig. 2:**
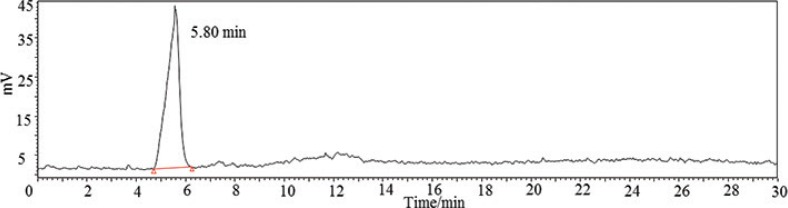
HPLC chromatogram showed the molecular weight of the EPS produced by *Lactobacillus coryniformis* NA-3, calculated by linear regression with dextran standards.

A chromatogram showing the uniformity of EPS-NA3 is shown in Fig. 2. The molecular weight was 8.6×10^6^ Da, calculated by linear regression with dextran standards (Log Mw = −0.7574 x + 11.328, R² = 0.9966, where Mw: the molecular weight, and x: retention time). Previous studies have found that the molecular mass of most HePSs is between 1×10^4^ Da and 6×10^6^ Da (4, 36, 37). For example, Cerning (9) has indicated that *S. salivarius* sp. *thermophilus* grown on skimmed milk could produce two fractions of EPS, one was close to 2×10^6^Da and another was 3.5×10^4^ Da. In this work, we found that the molecular weight of EPS-NA3 was basically comparable with previous reports, but was a bit more than the maximum molecular weight found in the literature.

The composition of EPS-NA3, extracted from batch cultures of NA-3 and subjected to HPLC analysis was determined. The yield of EPS was 6.9 mg/L. The phenol–sulfuric acid method revealed that the total sugar content of EPS was 61%. Acid hydrolysis of EPS followed by HPLC analysis (Supplementary Figs. S1 and S2) showed that the monosaccharide composition of EPS included rhamnose, mannose, galactose, and glucose. The molar ratio of four monomers Rha:Man:Gal:Glc was 2.6:1.0:5.0:3.3. The presence of different monomers indicated that the EPS was a HePS.

### FT-IR shows characteristic peaks for polysaccharide functional groups

**Fig. 3:**
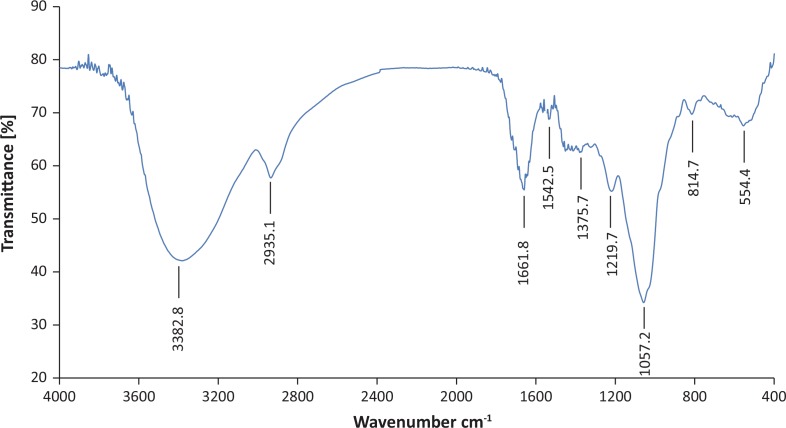
The FT-IR spectrum of exopolysaccharide (EPS) produced by *Lactobacillus coryniformis* NA-3 with the KBr pellet pressing method; *x*-axis is wave number (cm^−1^) and *y*-axis is transmittance (%).

In order to determine the characteristic structure of EPS, FT-IR spectroscopy of EPS-NA3 was conducted. The FT-IR spectrum of EPS-NA3 showed a complex pattern of peaks from 3000 cm^−1^ to 1000 cm^−1^ (Fig. 3). Specifically, several characteristic functional groups were revealed, such as a broad-stretching hydroxyl group at 3382.8 cm^−1^; a weak C-H stretching peak of methyl group at 2935.1 cm^−1^; a distinct peak corresponding to an amide I>C=O stretching and C-N bending of protein and peptide amines at 1661.8 cm^−1^; and a peak at 1375.7 cm^−1^ assigned to >C=O stretching of COO^−^ as well as a C-O band from the same COO^−^ group (27). The presence of characteristic hydroxyl groups suggested that EPS-NA3 was a polysaccharide. Furthermore, in the fingerprint region of polysaccharide, the main absorption bands were a sharp peak at 1057.2 cm^−1^ and a weak peak at 1219.7 cm^1^, which are typical of C-O (alcoholic hydroxyl group) and C-O-C groups (carbon–oxygen absorption peak on the ring) (38). Thus, FT-IR spectroscopy revealed that EPS-NA3 we extracted from NA-3 culture medium contained most of the characteristic absorption peaks associated with polysaccharides.

### NMR spectrum of EPS-NA3

**Fig. 4:**
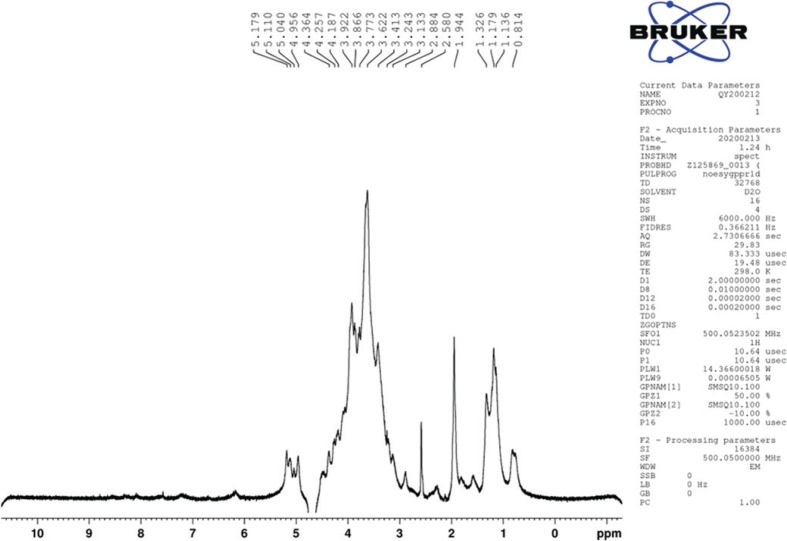
The^1^H NMR spectrum of EPS-NA3.

Hydrogen-1 Nuclear Magnetic Resonance (^1^H NMR) was always used to analyze the glycosidic bond configuration of polysaccharides (39). The EPS-NA3 was further characterized by one-dimensional ^1^H-NMR as shown in Fig. 4. It was the same as polysaccharide, the ^1^H NMR spectrum of EPS-NA3 also consisted of mainly three regions. The first region is anomeric proton region (δH 4.5–5.5 ppm), which is often used to differentiate the anomeric protons of sugar residues in polysaccharides; the second one is ring proton region (δH 3.1–4.5 ppm), a typical region for polysaccharides, including many crowded signals due to the presence of many sugar residues; and the last is the alkyl region (δH 1.2–2.3 ppm) (40, 41). Four major chemical shift signals (δ4.956, δ5.040, δ5.110, and δ5.179) in δ4.5–5.5 ppm were obtained in ^1^H NMR, suggesting that EPS-NA3 mainly contained four monosaccharide residues, corresponding to the existence of four monomers. Chemical shifts between 4.9 ppm and 5.3 ppm are typical of the anomeric protons of these α-linked residues, whereas β-anomeric protons usually resonate between 4.9 ppm and 5.3 ppm (39), certifying that all sugar residues in EPS-NA3 were linked by α-glycosidic bond. The signals of EPS-NA3 detected in the spectrum in δ3.2–4.4 were resolved with difficulty due to the protons attached to C2 and C6, resulting in the overlapping of chemical shifts (42). Results of ^1^H NMR spectrum were corresponding with the analysis of EPS-NA3 by HPLC and FT-IR, drawing a conclusion that EPS-NA3 was completely confirmed as polysaccharide. EPS-NA3 comprised α-mannose, α-glucose, α-galactose, and α-rhamnose residues.

### Antioxidant properties of EPS-NA3

#### Hydroxyl radical-scavenging property

**Fig. 5:**
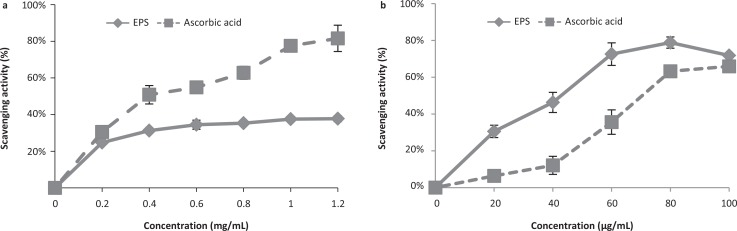
Free radical-scavenging activities of EPS derived from *Lactobacillus coryniformis* NA-3 and ascorbic acid (positive control): hydroxyl radicals (a) and superoxide radicals (b). Data are represented as mean ± standard deviation (SD) of three replicates per experiment. Three independent experiments were conducted for each assay.

Hydroxyl radicals are powerful oxidants that react with almost all biological molecules, including nucleic acids, proteins, lipids, and carbohydrates (11). The *in vitro* antioxidant activity of EPS-NA3 was evaluated by assaying its ability to scavenge hydroxyl radicals. The EPS exhibited concentration-dependent scavenging activity against hydroxyl radicals from 0.2 mg/mL to 1.2 mg/mL (Fig. 5a). The antioxidant activity of 0.2 mg/mL EPS (24.71% ± 0.83%) was similar to that of ascorbic acid (30.52% ± 3.16%) in the same concentration. In addition, scavenging increased with concentration, although ascorbic acid activity increased more rapidly than that of EPS. Eventually, the scavenging activity of EPS-NA3 (37.77% ± 1.56%) plateaued at 1.2 mg/mL, about half that of ascorbic acid (81.59 ± 7.18%), indicating that EPS-NA3 may serve as a good alternative to ascorbic acid, with a low antioxidant effect.

#### Superoxide radical-scavenging property

Superoxide radicals, singlet oxygen atoms, are active oxygen-free radicals produced in the human body that can trigger lipid peroxidation *in vivo* (43). In addition to hydroxyl radicals, we tested the ability of EPS-NA3 to neutralize superoxide in order to determine whether there were differences in its specificity for scavenging free radicals. The scavenging activity of EPS-NA3 was also concentration-dependent against superoxide radicals (Fig. 5b) generated by pyrogallol autoxidation. The scavenging capability of EPS was higher than that of ascorbic acid, with 20–60 μg/mL (30.55% ± 3.37%–72.59% ± 6.14%) of EPS-NA3 exhibiting roughly twice the antioxidant activity of ascorbic acid (6.38% ± 1.45%–35.61% ± 6.63%) at the same concentration. The activities of both EPS-NA3 and ascorbic acid gradually plateaued and the gap was reduced at concentrations higher than 80 μg/mL. However, the maximum superoxide scavenging activity of EPS (78.87% ± 3.07%) was higher than that of ascorbic acid (63.23% ± 1.54%) at the same concentration (80 μg/mL), thus indicating that this EPS is a more potent antioxidant against O_2_^−^than ascorbic acid.

### *In vitro* anti-biofilm activities of EPS-NA3

**Fig. 6:**
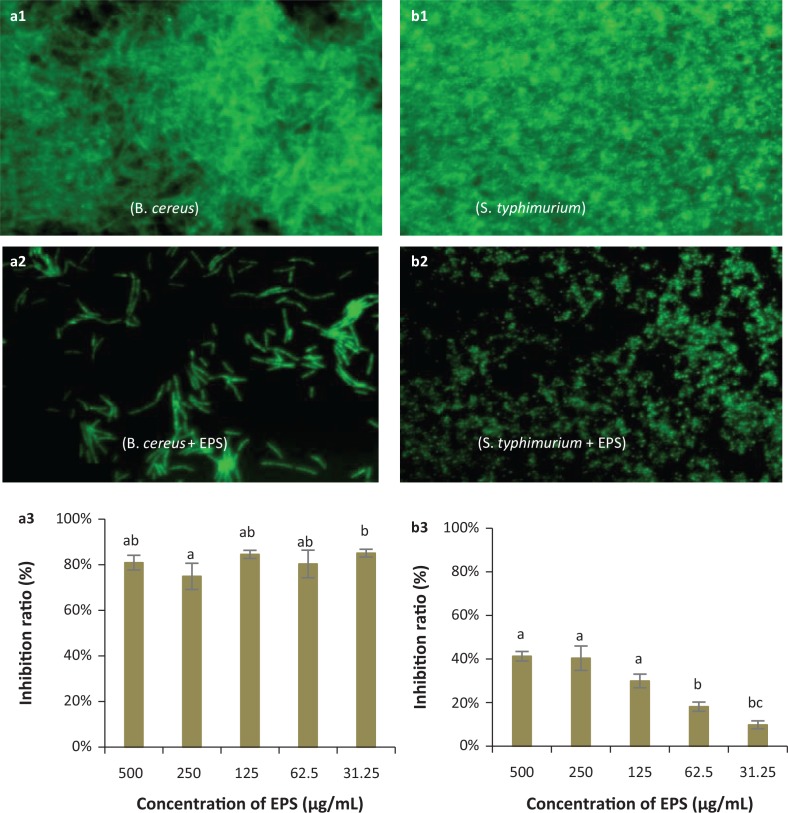
Representative micrographs (40×) showing the inhibitory effects of EPS on biofilm formation by pathogenic bacteria: *Bacillus cereus* (a1, a2, a3) and *Salmonella typhimurium* (b1, b2, b3) and spectrophotometric analyses of EPS biofilm inhibition. (a1, b1): Negative controls (pathogen only, no EPS) for biofilm formation by fluorescence microscopy; (a2, b2): Biofilm formation in the presence of EPS observed by fluorescence microscopy; (a3, b3): Inhibition ratios of EPS against *B. cereus* and *S. typhimurium* biofilms respectively. ^a,b,c^Different letters indicate significant differences between concentrations (*P* < 0.05).

Given its capacity for antioxidant activity, we next decided to test whether EPS-NA3 also exhibited the ability to inhibit biofilm formation (31), given the role of EPS in preventing colonization by competing bacteria. Observation by fluorescence microscopy of *B. cereus* (Figs. 6a1 and 6a2) and *S. typhimurium* (Figs. 6b1 and 6b2) biofilms that developed in the absence or presence of EPS-NA3, respectively, revealed that the density of biofilms was lower when the pathogens were cultured with EPS-NA3 compared with the control group, which was not exposed to EPS. The EPS-NA3 was, therefore, able to reduce biofilm formation by *B. cereus* and *S. typhimurium*. Inhibition ratios determined by measuring with UV spectrophotometry indicated that against *B. cereus* (Fig. 6a3) and *S. typhimurium* (Fig. 6b3) biofilms, EPS-NA3 had a strong ability for inhibition of *B. cereus* biofilm formation, although less so for *S. typhimurium*. The inhibition ratio reached 80%, and the effect was concentration-independent in *B. cereus* assays when the concentrations of EPS-NA3 were between 31.25 μg/mL and 500 μg/mL. Inhibition of *S. typhimurium* biofilm formation was also concentration-dependent, with inhibition ratios ranging from 9.71% ± 1.80% to 40.87% ± 2.2% with EPS concentrations between 31.25 μg/mL and 500 μg/mL respectively. Although EPS-NA3 successfully reduced biofilm formation by both *B. cereus* and *S. typhimurium,* it was more effective against *B. cereus* than *S. typhimurium.*

**Fig. 7:**
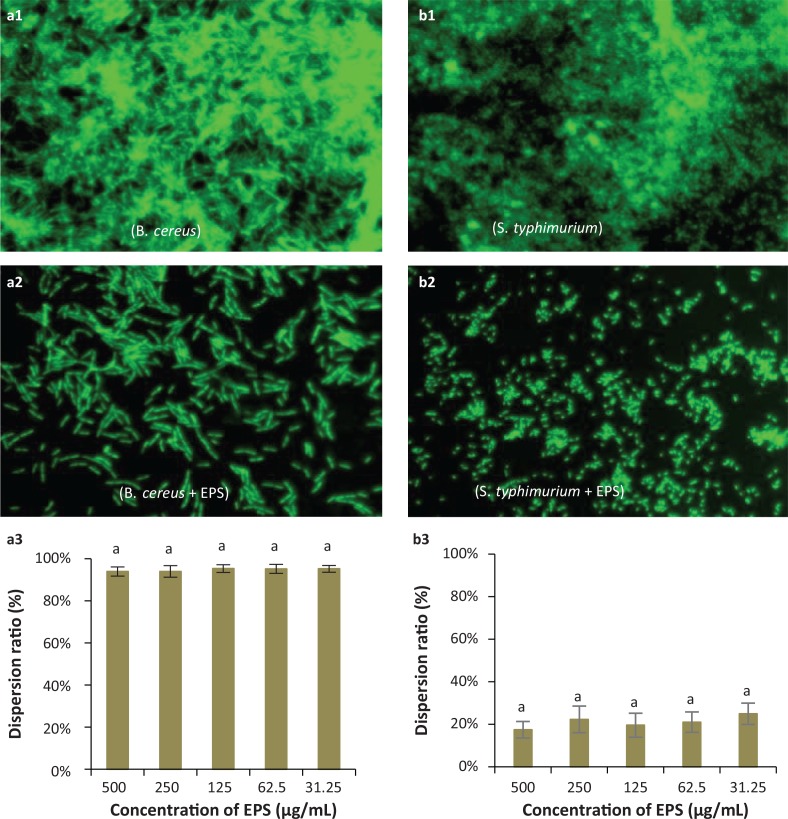
Representative micrographs (40×) showing the dispersion activity by EPS against established biofilms of the pathogenic bacteria *Bacillus cereus* (a1, a2, a3) and *Salmonella typhimurium* (b1, b2, b3), and spectrophotometric analyses of EPS-mediated dispersion of biofilms produced by these species. (a1, b1): Observation by fluorescence microscopy of negative controls (pathogens only, no EPS) for biofilm dispersion; (a2, b2): Observation by fluorescence microscopy of biofilm dispersion by *Lactobacillus coryniformis* NA-3-derived EPS; (a3, b3): Dispersion ratios of EPS on different bacterial biofilms. ^a,b,c^Different letters indicate significant differences between treatments (*P* < 0.05).

To explore further the anti-biofilm activity of EPS-NA3, we also exposed fully formed biofilms to EPS to observe any potential dispersant activity. Fluorescence microscopy showed that EPS-NA3 exerts a dispersive effect on *B. cereus* (Figs. 7a1 and 7a2) and *S. typhimurium* (Figs. 7b1 and 7b2) biofilms. Dispersion ratios determined with spectrophotometry (Figs. 7a3 and 7b3) indicated that as much as 90% of *B. cereus* biofilm was dispersed by EPS-NA3, independent of concentration (range from 31.25 μg/mL to 500 μg/mL), while only approximately 20% of the *S. typhimurium* biofilm dispersed in the presence of EPS-NA3 (also independent of concentration). These data show that EPS-NA3 is effective for inhibiting the formation of biofilms as well as for removing mature biofilms, although more effectively against *B. cereus* than against *S. typhimurium.*

## Discussion

EPS-producing *Lactobacillus* spp. have long been established as an integral component in the production of many foods worldwide, and in that capacity they have been classified as ‘generally recognized as safe (GRAS)’. LAB-derived EPSs are high molecular weight, long-chain, linear or branched biopolymers secreted to the external environment or adhered to the bacterial cell surface (44). Owing to their reported probiotic characteristics, such as anti-tumor, immune-stimulating, antioxidant, and anti-biofilm activities, EPSs produced by LAB have become the focus of research across several disciplines, spanning pharmaceutical development, cancer biology, food science, and industrial engineering.

In this study, we isolated the EPS-producing *Lactobacillus* sp. *L. coryniformis* NA-3 from northeastern Chinese sauerkraut (Suan Cai), which is rich in a variety of potentially probiotic microorganisms. Many EPS-producing LAB have been previously screened from a variety of fermented foods such as milk and fermented cabbage (3, 20). However, to our knowledge, this work represents the first report characterizing EPS from *L. coryniformis*. As significant components of the cell surface, exopolysaccharides play a critical role in mediating cell-to-cell interactions (45). LAB-derived EPSs produced *in vitro* can exhibit antimicrobial properties that have been proposed to act by binding to biofilm-related signal molecules or glycocalyx receptors on the surfaces of pathogen cells. This receptor binding subsequently disrupts communication between cells, thereby interfering with the formation of biofilms, and eventually leading to the inhibition of pathogen growth and proliferation (12). We extracted EPS from *L. coryniformis* NA-3 for analysis of its compositions and physico-chemical characteristics, and subsequently found evidence of its antioxidant ability and biofilm-inhibiting activity.

Polysaccharides are soluble in water but not in alcohol, so we used a hot water-based extraction followed by alcohol precipitation to isolate EPS, and then added TCA for deproteinization. In this work, we observed that EPS yields were very low (6.9 mg/mL), possibly due to culture of NA-3 in MRS broth. Although there are no reports about EPS yield from *L. coryniformis*, previous works have shown that EPS production from other strains is affected by many factors such as carbon source (46), composition of medium, temperature, and pH (4). Studies have also suggested that carbon source is a significant factor that promoted EPS production and high EPS could be produced in the presence of sucrose (39, 47). Actually, it has been proved that the EPS production could be improved when strain was grown in the optimal incubation conditions compared to incubating in a control grown on commercial MRS medium (48). Therefore, *L. coryniformis* NA-3 might produce higher levels of EPS after optimization of culture conditions in the future.

FT-IR spectroscopy and ^1^H NMR spectrum analysis of the extracted EPS showed absorption peaks characteristic of sugar, and acid hydrolysis followed by HPLC revealed that EPS-NA3 was a HePS composed of α-rhamnose, α-mannose, α-galactose, and α-glucose. Previous studies have shown that EPS isolated from *L. casei* LC2W is composed of glucose, rhamnose, and galactose (20). Similarly, the EPS from *L. plantarum* was found to contain three monosaccharides: mannose, glucose, and galactose (49). Recent researches have also reported EPS extracted from another *L. plantarum* composed of glucose, galactose, and fructose (39), and a new EPS from *Lactobacillus fermentum* YL-11, including four different monosaccharides (galactose, glucose, mannose, and arabinose). Although glucose, mannose, and galactose are always present in most of EPSs, composition of EPS monomers varies from strain to strain.

The free radical theory of aging proposes that essential biological processes become disrupted with increasing age, eventually leading to serious illnesses such as diabetes and Alzheimer’s disease (50). Therefore, studies exploring free radical-scavenging of natural antioxidants, such as those produced by LAB, can lead to safe and effective medicines that inhibit the progression of chronic diseases (32). Gomaa and Yousef (47) have suggested that the EPS from *Virgibacillus salarius* BM02 was able to scavenge hydroxyl radicals and the scavenging activity reached 60.00% ± 0.06% at 10 mg/mL. However, studies of EPS from *Pseudomonas aeruginosa* exhibited better scavenging activity on both hydroxyl and superoxide radicals: it could scavenge both hydroxyl radicals up to 50% at a concentration of 60 μg/mL and superoxide radicals up to 70% at a concentration of 60 μg/mL (31). In our examination of the antioxidant properties of *L. coryniformis* EPS-NA3, we found that this EPS could scavenge hydroxyl radicals and superoxide radicals. In comparison, the maximum scavenging activity of *L. coryniformis* EPS-NA3 on hydroxyl radicals is lower than the EPS from *Virgibacillus salarius* BM02, but the former (around 35%) is better than the latter (less than 10%) when they worked at the same concentration of 1 mg/mL. On the other hand, the scavenging activity of *L. coryniformis* EPS-NA3 on hydroxyl radicals is less than the EPS isolated from *Pseudomonas aeruginosa*; however, our EPS showed better ability on superoxide radicals (72%) at a concentration of 60 μg/mL. Interestingly, the scavenging activity of *L. coryniformis* EPS-NA3 on hydroxyl and superoxide radicals varies greatly with concentrations, showing a better ability to scavenge superoxide radicals than hydroxyl radicals. Prior studies have indicated that EPS can reduce levels of cell-damaging free radicals and oxidizing agents (15) but is affected by molecular weight, composition, degree of polymerization, and number of side chains of EPS (33). Hence, the structural differences are most likely to be responsible for different scavenging activities.

In addition to antioxidant ability, we also focused on the ability of EPS-NA3 to mitigate the formation of biofilms and to reduce established biofilms. Biofilms are surface-attached extracellular matrices composed of a complex of nucleic acids, proteins, polysaccharides, and lipids. The polysaccharide components play significant roles as virulence factors that mediate pathogenesis or as essential signals in host-pathogen interactions (46, 49). The formation of biofilms by pathogenic bacteria can pose a serious public health risk by decreasing bacterial susceptibility to antimicrobial agents and thus improving their defense mechanisms (51). Previous research has demonstrated that many EPSs from *Lactobacillus spp*. can decrease biofilm formation by pathogens or disperse established biofilms, thereby exhibiting antibacterial activities. EPS isolated from *L. plantarum* showed anti-biofilm activity on the biofilm of *P. aeruginosa, S. typhimurium, Staphylococcus aureus*, and *Listeria monocytogenes* at a concentration of 256 μg/mL and found that dispersion effects are better than inhibition (17). Moreover, EPS from *Lactobacillus fermentum* S1 had a favorable anti-biofilm activity against *Escherichia coli* and *Staphylococcus aureus* and the highest inhibition ratios are 32% and 43% respectively (52). Previous studies have demonstrated that EPS from *L. plantarum* YW32 and *L. acidophilus* A4 were found to possess anti-biofilm activity on both Gram-negative and Gram-positive bacteria (19, 53). In this study, we observed that pathogen-produced biofilms decreased when cultured in the presence of EPS-NA3 solution, indicating that EPS played a critical role in attenuating biofilm formation. EPS-NA3 showed a high inhibition and dispersion ratios on *B. cereus* (Gram-positive) biofilm, reaching 80% and 90% respectively. However, no significant effect was observed between 31.25 and 500 μg/mL of EPS-NA3 (*P* > 0.05). Obviously, both inhibition and dispersion ratios on *B. cereus* in presence of EPS-NA3 were much higher than the above-reported ratios, suggesting that EPS-NA3 is a highly effective biofilm inhibitor on *B. cereus*. In contrast, biofilm inhibition against *S. typhimurium* was concentration-dependent; higher concentration led to higher inhibitory efficacy, with a maximum inhibition of 40% at an EPS concentration of 500 μg/mL. The dispersion ratio was about 20% for *S. typhimurium* and the level of activity was independent of dosage. These results indicated that EPS-NA3 also worked on some biofilms formed by Gram-negative bacteria in agreement with the previous experimental results. Overall, EPS-NA3 had a stronger effect against the biofilm of *B. cereus* than against that of *S. typhimurium.*

## Conclusion

The EPS extracted from *L. coryniformis* NA-3, screened from northeastern Chinese sauerkraut (Suan Cai), is a HePS comprising α-rhamnose, α-mannose, α-galactose, and α-glucose, with antioxidant and anti-biofilm properties. Based on the results of this study, we conclude that EPS-NA3 can scavenge free radicals, especially superoxide radicals, inhibit biofilm formation, and disperse the biofilms of *B. cereus* and *S. typhimurium*. Additional research on EPS-NA3 could further characterize the mechanisms underlying its activity and optimize its production and composition for potential applications as an antioxidant and anti-biofilm agent in food and pharmaceutical industries.

## Supplementary Material

A novel exopolysaccharide produced by *Lactobacillus coryniformis* NA-3 exhibits antioxidant and biofilm-inhibiting properties *in vitro*
