# Supplementary material for: A novel exopolysaccharide produced by Lactobacillus coryniformis NA-3 exhibits antioxidant and biofilm-inhibiting properties in vitro
Source: Food Nutr Res. 2020 Apr 3;64:10.29219/fnr.v64.3744. doi: 10.29219/fnr.v64.3744 (PMC7217292; doi:10.29219/fnr.v64.3744)
Supplement: A novel exopolysaccharide produced by Lactobacillus coryniformis NA-3 exhibits antioxidant and biofilm-inhibiting properties in vitro [file FNR-64-3744-s001.docx]

**Supplementary figures:**

**Figure S1:** HPLC chromatogram of the standard monosaccharides，including rhamnose, arabinose, mannose, galactose and glucose. The retention time of each monosaccharide is 7.90 min, 10.5 min, 17.5 min, 19.0 min and 20.6 min, respectively.

**Figure S2:** HPLC chromatogram showing the monosaccharide composition of the EPS produced by *Lactobacillus coryniformis* NA-3 by comparing the retention time with the standard monosaccharides.

**Time/min**

**mV**

**rhamnose**

**mannose**

**glucose**

**galactose**

**mV**

**Time/min**

**mannose**

**galactose**

**glucose**

**rhamnose**

**arabinose**
